# Recent advances in basic neurosciences and brain disease: from synapses to behavior

**DOI:** 10.1186/1744-8069-2-38

**Published:** 2006-12-30

**Authors:** Guo-Qiang Bi, Vadim Bolshakov, Guojun Bu, Catherine M Cahill, Zhou-Feng Chen, Graham L Collingridge, Robin L Cooper, Jens R Coorssen, Alaa El-Husseini, Vasco Galhardo, Wen-Biao Gan, Jianguo Gu, Kazuhide Inoue, John Isaac, Koichi Iwata, Zhengping Jia, Bong-Kiun Kaang, Mikito Kawamata, Satoshi Kida, Eric Klann, Tatsuro Kohno, Min Li, Xiao-Jiang Li, John F MacDonald, Karim Nader, Peter V Nguyen, Uhtaek Oh, Ke Ren, John C Roder, Michael W Salter, Weihong Song, Shuzo Sugita, Shao-Jun Tang, Yuanxiang Tao, Yu Tian Wang, Newton Woo, Melanie A Woodin, Zhen Yan, Megumu Yoshimura, Ming Xu, Zao C Xu, Xia Zhang, Mei Zhen, Min Zhuo

**Affiliations:** 1 Department of Neurobiology, University of Pittsburgh, Pittsburgh, USA; 2 Department of Psychiatry, Harvard University, Boston, USA; 3 Department of Pediatrics, and Cell Biology and Physiology, Washington University in St. Louis, St. Louis, USA; 4 Department of Pharmacology and Toxicology, Queen's University, Kingston, Canada; 5 Department of Anesthesiology, Washington University in St. Louis, St. Louis, USA; 6 Centre for Synaptic Plasticity, University of Bristol, Bristol, UK; 7 Department of Biology, University of Kentucky, Lexington, USA; 8 Department of Physiology and Biophysics, University of Calgary, Calgary, Canada; 9 Department of Psychiatry, University of British Columbia, Vancouver, Canada; 10 Institute for Molecular and Cell Biology, University of Porto, Porto, Portugal; 11 Skirball Institute, New York University School of Medicine, New York, USA; 12 Department of Oral and Maxillofacial Surgery, University of Florida, Gainesville, USA; 13 Department of Pharmaceutical Health Care and Sciences, Kyushu University, Kyushu, Japan; 14 NINDS, NIH, Bethesda, USA; 15 Department of Physiology, Nihon University, Tokyo, Japan; 16 Department of Physiology, University of Toronto, Toronto, Canada; 17 Department of Biological Sciences, Seoul National University, Seoul, Korea; 18 Department of Anesthesiology, Sapporo Medical University School of Medicine, Sapporo, Japan; 19 Department of Agricultural Chemistry, Tokyo University of Agriculture, Tokyo, Japan; 20 Department of Molecular Physiology and Biophysics, Baylor College of Medicine, Houston, USA; 21 Division of Anesthesiology, Niigata University, Niigata, Japan; 22 Department of Neuroscience and High Throughput Biology Center, Johns Hopkins University, Baltimore, USA; 23 Department of Human Genetics, Emory University, Atlanta, USA; 24 Department of Psychology, McGill University, Montreal, Canada; 25 Department of Physiology, University of Alberta, Edmonton, Canada; 26 Sensory Research Center, Seoul National University, Seoul, Korea; 27 Department of Biomedical Sciences, University of Maryland, Baltimore, USA; 28 Department of Medical Genetics and Microbiology, University of Toronto, Toronto, Canada; 29 Department of Neurobiology and Behavior, University of California, Irvine, USA; 30 Department of Anesthesiology and Critical Care Medicine, Johns Hopkins University, Baltimore, USA; 31 Brain Research Center, University of British Columbia, Vancouver, Canada; 32 NICHD, NIH, Bethesda, USA; 33 Department of Cell and Systems Biology, University of Toronto, Toronto, Canada; 34 Department of Physiology and Biophysics, State University of New York at Buffalo, Buffalo, USA; 35 Department of Basic Medicine, Kyushu University, Kyushu, Japan; 36 Department of Anesthesia and Critical Care, University of Chicago, Chicago, USA; 37 Department of Anatomy and Cell Biology, Indiana University School of Medicine, Indianapolis, USA; 38 Department of Psychiatry, University of Saskatchewan, Saskatoon, Canada

## Abstract

Understanding basic neuronal mechanisms hold the hope for future treatment of brain disease. The 1st international conference on synapse, memory, drug addiction and pain was held in beautiful downtown Toronto, Canada on August 21–23, 2006. Unlike other traditional conferences, this new meeting focused on three major aims: (1) to promote new and cutting edge research in neuroscience; (2) to encourage international information exchange and scientific collaborations; and (3) to provide a platform for active scientists to discuss new findings. Up to 64 investigators presented their recent discoveries, from basic synaptic mechanisms to genes related to human brain disease. This meeting was in part sponsored by Molecular Pain, together with University of Toronto (Faculty of Medicine, Department of Physiology as well as Center for the Study of Pain). Our goal for this meeting is to promote future active scientific collaborations and improve human health through fundamental basic neuroscience researches. The second international meeting on Neurons and Brain Disease will be held in Toronto (August 29–31, 2007).

## Introduction

One key factor to promote the progress of science is to exchange scientific ideas and new discovery through meetings. Scientific meeting provides critical chance for investigators to communicate new ideas, discuss different/conflicting results, and set up potential collaborations. Annual meeting of the American Society for Neuroscience (SfN) has served the community well in this aspect. However, with the increased membership and the scale of the meeting, SfN meetings only take place in a few cities in US in a rotated manner. Due to tight security control after 9/11, many foreign investigators failed to obtain visiting visa to the meeting in a timely fashion. Considering these factors, a small scale of neuroscience meeting in a more relaxed city should provide better chance for investigators, in particular, principal investigators (PIs) to directly communicate with each other, face-to-face. The 1st international conference on synapse, memory, drug addiction and pain is designed to meet the need. The major aim of this meeting is to provide an opportunity for setting up global scientific exchanges, to provide an active stage for PIs to report novel or unpublished data, to bring neuroscientists working at different level of organism and systems together, and to promote research findings from junior and mid-career investigators.

The meeting is organized by Dr. Min Zhuo from the University of Toronto, with the help from Dr. Jianguo Gu from the University of Florida, and in part sponsored by Molecular Pain, University of Toronto (Faculty of Medicine, Department of Physiology as well as Center for the Study of Pain), and Olympus Inc.

There are four major themes for the meeting: synapse, Synaptic plasticity, memory, pain, and brain disease. Unlike other meetings, each speaker was given 15 min to talk, and 5 min for discussion. The time slot allowed for each speaker was tightly controlled by each chair (with a timer!), and few speakers went beyond the time permitted. The wine reception over the poster section provided a wonderful opportunity for further discussions. Taking advantage of the excellent location of the main campus in the downtown of Toronto, attendees also enjoyed the nice summer weather and wonderful food in Toronto. The Niagara Fall, a famous tour site, is above 90 min drive from the downtown of Toronto.

Students and post-doc fellows also presented over 50 posters on various topics. Among them, three posters were selected for best poster presentations and have been awarded with $250–500.

## Text

### List of major themes, chairs, speakers and titles

#### Synapse

Chair: Min Li (USA)

• Jens R Coorssen (University of Calgary, Calgary, Canada)

The role of cholesterol in synaptic release

• Lin Mei (Medical College of Georgia, Augusta, USA)

Neuregulin regulation of neuronal activity

• Alaa El-Husseini (University of British Columbia, Vancouver, Canada)

Mechanisms that govern protein assembly at nascent neuronal contacts

• Wen-Biao Gan (New York University School of Medicine, New York, USA)

Dendritic Spine Stability and Its Modification by Experience

• Elise F Stanley (Toronto Western Research Institute, Toronto, Canada)

The presynaptic transmitter release site complex

Chair: Alaa El-Husseini (Canada)

• Mei Zhen (University of Toronto, Toronto, Canada)

SAD kinase regulates neuronal polarity and synapse formation

• Robin L Cooper (University of Kentucky, Lexington, USA)

Effects of the serotonergic system on physiology, development, learning and behavior of drosophila melanogaster

• Min Li (Johns Hopkins University, Baltimore, USA)

Chemical regulation of membrane excitability

• Shuzo Sugita (University of Toronto, Toronto, Canada)

Molecular mechanism of GTP-dependent exocytosis

• Lu-Yang Wang (University of Toronto, Toronto, Canada)

Convergent pre- and post-synaptic adaptations for high-fidelity neurotransmission at the developing calyx of Held synapse

• Wei-Yang Lu (University of Toronto, Toronto, Canada)

Physical Interaction between Acetylcholinesterase and Neurexins

#### Synaptic plasticity

Chair: Graham L Collingridge (UK) and Yu Tian Wang (Canada)

• Eric Klann (Baylor College of Medicine, Houston, USA)

Translational Control during Hippocampal Synaptic Plasticity and Memory

• Peter V Nguyen (University of Alberta, Edmonton, Canada)

Beta-Adrenergic Receptors Recruit ERK and mTOR to Promote Translation-Dependent Synaptic Plasticity

• Shao-Jun Tang (University of California, Irvine, USA)

Regulation of Activity-Dependent Protein Synthesis in Dendrites

• Yu Tian Wang (University of British Columbia, Vancouver, Canada)

Synaptic plasticity in learning and memory

• Graham L Collingridge (University of Bristol, Bristol, UK)

Glutamate receptors and synaptic plasticity in the hippocampus

• Michael W Salter (University of Toronto, Toronto, Canada)

Ins and outs of SRC regulation of NMDA receptors and synaptic plasticity

• John F MacDonald (University of Toronto, Toronto, Canada)

Inhibitory Regulation of the Src Hub and LTP in CA1 Hippocampal Neurons

Chair: Michael W Salter (Canada)

• Vadim Bolshakov (Harvard University, Boston, USA)

Spatiotemporal asymmetry of associative synaptic plasticity in fear conditioning pathways

• Guo-Qiang Bi (University of Pittsburgh, Pittsburgh, USA)

Dynamics and plasticity of reverberatory activity in small neuronal circuits

• Melanie A Woodin (University of Toronto, Toronto, Canada)

Bidirectional spike-timing dependent plasticity of inhibitory transmission in the hippocampus

• John Isaac (NIH, Bethesda, USA)

Kainate receptors in novel forms of long-term synaptic plasticity

• Newton Woo (NIH, Bethesda, USA)

Regulation of Bi-directional Plasticity by BDNF

• Zhengping Jia (University of Toronto, Toronto, Canada)

Molecular regulation of spine properties and synaptic plasticity

#### Pain

**C**hair: Megumu Yoshimura (Japan)

• Kazuhide Inoue (Kyushu University, Kyushu, Japan)

P2X4: mechanisms of over expression in neuropathic pain state

• Jianguo Gu (University of Florida, Gainesville, USA)

TRPM8 and cold allodynia

• Uhtaek Oh (Seoul National University, Seoul, Korea)

TRPV1 and its Role for Inflammatory Pain

• Vasco Galhardo (University of Porto, Porto, Portugal)

Impairment in prefrontal-based emotional decision-making in rat models of chronic pain

• Ke Ren (University of Maryland, Baltimore, USA)

Neuronal/glial cell interactions in CNS plasticity and persistent pain

Chair: Jianguo Gu (USA)

• Yves De Koninck (Laval University, Quebec City, Canada)

Plasticity of chloride homeostasis vs. plasticity of GABA/glycine; who wins?

• Megumu Yoshimura (Kyushu University, Kyushu, Japan)

Synaptic mechanisms of acupuncture in the spinal dorsal horn revealed by in vivo patch-clamp recordings

• Koichi Iwata (Nihon University, Tokyo, Japan)

Anterior cingulate cortex and pain -its morphological feature and functional properties

• Min Zhuo (University of Toronto, Toronto, Canada)

Cortical potentiation and its roles in persistent pain and fear

• Vania A Apkarian (Northwestern University, Chicago, USA)

Chronic pain and emotional learning and memory

Chair: Uhtaek Oh (Korea)

• Zhou-Feng Chen (Washington University in St. Louis, St. Louis, USA)

Living without serotonin: a genetic approach to study the roles of the serotonergic system in opioid analgesia and tolerance

• Catherine M Cahill (Queen's University, Kingston, Canada)

Trafficking of Delta Opioid Receptors in Chronic Pain

• Hiroshi Ueda (Nagasaki University, Nagasaki, Japan)

Molecular mechanisms of neuropathic pain – lysophosphatidic acid as the initiator

• Yuanxiang Tao (Johns Hopkins University, Baltimore, USA)

Are the PDZ domains at excitatory synapses potential molecular targets for prevention and treatment of chronic pain?

• Tatsuro Kohno (Niigata University, Niigata, Japan)

Different actions of opioid and cannabinoid receptor agonists in neuropathic pain

• Ze'ev Seltzer (University of Toronto, Toronto, Canada)

Power and limitations of the comparative approach that uses animal models to identify human chronic pain genes

• Mikito Kawamata (Sapporo Medical University School of Medicine, Sapporo, Japan)

Genetic variation in response properties of spinal dorsal horn neurons and rostral ventromedial medulla neurons in different mouse strains

#### Brain disease

Chair: Xiao-Ming Xu (USA)

• Guojun Bu (Washington University in St. Louis, St. Louis, USA)

LDL Receptor Family and Alzheimer's disease

• Satoshi Kida (Tokyo University of Agriculture, Tokyo, Japan)

Mechanism of interaction between reconsolidation and extinction of contextual fear memory

• Weihong Song (University of British Columbia, Vancouver, Canada)

Hypoxia facilitates Alzheimer's disease pathogenesis

• Zhen Yan (State University of New York at Buffalo, Buffalo, USA)

Interactions between Acetylcholine, Amyloid and Ion Channels in Alzheimer's Disease

• Jian Feng (State University of New York at Buffalo, Buffalo, USA)

Achilles' Heel of Midbrain Dopaminergic Neurons: Vulnerabilities and Defense Strategies

• Xiao-Jiang Li (Emory University, Atlanta, USA)

Synaptic toxicity of Huntington disease protein

Chair: Xiao-Jiang Li (USA)

• Fang Liu (University of Toronto, Toronto, Canada)

Regulation of dopamine reuptake by the direct protein-protein interaction between the dopamine D2 receptor and the dopamine transporter

• Danny G Winder (Vanderbilt University School of Medicine, Nashville, USA)

Synaptic plasticity in the bed nucleus of the stria terminalis: roles in addiction and anxiety

• Ming Xu (University of Chicago, Chicago, USA)

Molecular Mechanisms of neuronal plasticity induced by drugs of abuse

• Xia Zhang (University of Saskatchewan, Saskatoon, Canada)

TAT-3L4F, a novel peptide for the treatment of drug addiction

• Evelyn K Lambe (University of Toronto, Toronto, Canada)

Hypocretin and nicotine excite the same thalamocortical synapses in prefrontal cortex: correlation with improved attention in rat

• Wan Qi (University of Toronto, Toronto, Canada)

Regulation of NMDA and GABA-A receptors by the tumor suppressor PTEN

#### Memory

Chair: Karim Nader (Canada)

• Paul W Frankland (University of Toronto, Toronto, Canada)

Functional integration of adult-born granule cells into spatial memory networks in the dentate gyrus

• Mara Dierssen (Centre for Genomic Regulation, Barcelona, Spain)

Dendritic pathology and altered structural plasticity in Down syndrome: In the search of candidate genes

• Sheena A Josselyn (University of Toronto, Toronto, Canada)

Neuronal memory competition: The role of CREB

• Bong-Kiun Kaang (Seoul National University, Seoul, Korea)

Role of a novel nucleolar protein ApLLP in synaptic plasticity and memory in Aplysia

• Remi Quirion (Douglas Hospital Research Centre and INMHA, Montreal, Canada)

Novel genes possibly involved in learning and memory

Chair: Bong-Kiun Kaang (Korea)

• John C Roder (University of Toronto, Toronto, Canada)

Forward and reverse genetic screens in the mouse for mutants impaired in learning and memory

• Karim Nader (McGill University, Montreal, Canada)

Identifying the neural mechanisms by which boundary conditions inhibit reconsolidation from occurring.

• Yukio Komatsu (Nagoya University, Nagoya, Japan)

Role of BDNF in the production of LTP at visual cortical inhibitory synapses

• Xiao-Ming Xu (University of Louisville School of Medicine, Louisville, USA)

Spinal cord injury repair: combinatorial strategies involving neuroprotection and axonal regeneration

• Zao C Xu (Indiana University School of Medicine, Indianapolis, USA)

Synaptic plasticity in pathological conditions

## Abstracts

• **Guo-Qiang Bi (Department of Neurobiology, University of Pittsburgh, Pittsburgh, USA) – Dynamics and plasticity of reverberatory activity in small neuronal circuits**

The concept of cell assembly was proposed by Hebb to provide an elementary structure for thought process and memory. The Hebbian cell assembly has two essential properties: 1. neuronal activity can reverberate in specific sequences within the assembly without sustained external drive; 2. synaptic modification resulted from the reverberatory activity further stabilizes the reverberation. Using whole-cell patch-clamp recording and simultaneous calcium imaging, we found that brief (e.g. 1-ms) stimulation of few neurons in a small network of about 100 cultured hippocampal neurons could trigger reverberatory activity in the network lasting for seconds. Such reverberatory activity consists of repeating motifs of specific patterns of population activation in the network. Paired-pulse stimuli with inter-pulse interval of ~200–400 ms are more effective in activating such oscillatory reverberation. Furthermore, repeated activation of reverberation with paired-pulse stimuli leads to long-term enhancement of subsequent activation by single stimuli. In addition, pairing a non-effective input (that does not activate network reverberation) into one neuron with an effective input (that activates reverberation) into another can convert the non-effective pathway into an effective one. Reverberatory circuits in vitro may serve as a prototype of Hebbian cell assembly for studies of the dynamics properties and underlying cellular mechanisms. (Supported by NIMH and Burroughs Wellcome Fund)

• **Vadim Bolshakov (Department of Psychiatry, Harvard University, Boston, USA) – Spatiotemporal asymmetry of associative synaptic plasticity in fear conditioning pathways**

Long-term potentiation (LTP) in afferent inputs to the amygdala serves an essential function in the acquisition of fear memory. The factors underlying input specificity of synaptic modifications implicated in the information transfer in fear conditioning pathways remain unknown. We now show that synapses in two auditory inputs converging on the same LA neuron utilize a form of the temporally asymmetric learning rule when the strength of naïve synapses is only modified when a postsynaptic action potential closely follows the synaptic response. The stronger inhibitory drive in thalamic pathway, as compared to cortical input, hampers the induction of LTP at thalamo-amygdala synapses contributing to the spatial specificity of LTP in convergent inputs. These results indicate that spike timing-dependent synaptic plasticity in the LA is both temporarily and spatially asymmetric, which may contribute to the conditioned stimulus discrimination during fear behavior.

• **Guojun Bu (Department of Pediatrics, and Cell Biology and Physiology, Washington University in St. Louis, St. Louis, USA) – LDL Receptor Family and Alzheimer's Disease**

Amyloid-β peptide (Aβ) production and accumulation in the brain is a central event in the pathogenesis of Alzheimer's disease (AD). Recent studies have shown that apolipoprotein E (apoE) receptors, members of the low-density lipoprotein receptor (LDLR) family, modulate Aβ production as well as Aβ cellular uptake. Aβ is derived from proteolytic processing of amyloid precursor protein (APP), which interacts with several members of the LDLR family. Studies from our laboratory have focused on three members of the LDLR family, the LDLR-related protein (LRP), LRP1B, and the LDLR. Our *in vitro *cellular studies have shown that while LRP's rapid endocytosis facilitates APP endocytic trafficking and processing to Aβ, LRP1B's slow endocytosis inhibits these processes. In addition to modulating APP endocytic trafficking, LRP's rapid endocytosis also facilitates Aβ cellular uptake by binding to Aβ either directly or via LRP ligands such as apoE. Our *in vivo *studies using transgenic approach have shown that overexpression of LRP in CNS neurons increases cell-associated Aβ and this increase correlates with an enhanced memory deficits in mice. We are currently investigating the cellular mechanisms by which LRP facilitates intraneuronal Aβ accumulation, a pathological event that directly contributes to the early cognitive deficits seen in AD. Our preliminary results indicate that apoE plays an important role in intraneuronal Aβ accumulation, likely by shuttling Aβ into neurons via LRP-mediated pathways. We hypothesize that depending on the Aβ species (Aβ 40 vs. Aβ 42), its aggregation states (monomers vs. oligomers), and the presence of apoE isoforms (apoE3 vs. apoE4), at least a portion of Aβ that is internalized via an LRP-dependent pathway accumulates inside neurons. Molecular and cellular models underlying the mechanisms of LRP's involvements in AD will be presented and discussed.

• **Catherine M Cahill (Department of Pharmacology and Toxicology, Queen's University, Kingston, Canada) – Trafficking of Delta Opioid Receptors in Chronic Pain**

Neuropathic (NP) pain is defined as pain caused by a peripheral and/or central nervous system lesion with sensory symptoms and signs and is estimated to affect more than 1.5% of Americans. Despite its prevalence and adverse impact on functionality and quality of life, it remains a significant challenge for physicians as it is typically refractory to traditional analgesics. However research increasingly suggests a therapeutic role of δOR agonists in treating chronic pain. Our research aims to understand the changes in δOR expression and function using both *in vivo *and *in vitro *techniques in an animal model of NP pain. NP, but not sham-operated, rats developed cold- and thermal-hyperalgesia as well as tactile allodynia. Intrathecal administration of a selective δOR agonist significantly alleviated these nociceptive behaviours and these effects were attenuated by a selective δOR antagonist. Real-time RT-PCR and western blotting experiments revealed no change in overall expression of δOR in the dorsal spinal cord however preliminary studies suggest that induction of NP pain may induce changes in subcellular localization of δORs leading to enhanced analgesia.

• **Zhou-Feng Chen (Department of Anesthesiology, Washington University in St. Louis, St. Louis, USA) – Living without serotonin: a genetic approach to study the roles of the serotonergic system in opioid analgesia and tolerance**

Narcotics have long been used as an effective treatment for pain. The roles of the serotonergic (5-HT) system in opioid analgesia and tolerance, however, have been controversial. We have recently shown that the transcription factor Lm × 1b is essential for the development of 5-HT neurons. In the absence of Lm × 1b, all 5-HT neurons failed to develop in the raphe system. Because Lm × 1b-null mice die around birth, we have designed to strategy to delete Lm × 1b in 5-HT neurons only. Lm × 1b conditional knockout (CKO) mice lack all 5-HT neurons in the raphe system. Surprisingly, Lm × 1b CKO mice survive to the adulthood without motor deficiency. To assess the roles of the 5-HT system in opioid analgesia, we have examined the tail-flick responses of Lm × 1b CKO mice injected with mu-, kappa- and delta-opioid receptor agonists. In addition, we also examined the site of action of opioid receptor agonists by systemic, intrathecal and intracerebroventricular injections. These pharmacological studies revealed that the 5-HT system contributes differentially to opioid analgesia. Moreover, an examination of morphine analgesic tolerance in Lm × 1b CKO mice indicated that morphine tolerance is independent of the 5-HT system. These results should have important implications in our understanding of mechanisms of action for the 5-HT system in opioid analgesia and tolerance.

• **Graham L Collingridge (Centre for Synaptic Plasticity, University of Bristol, Bristol, UK) – Kainate receptors: Functions and the discovery of novel antagonists.**

Less is known about the role of kainate receptors compared with the other classes of ionotropic glutamate receptors in the CNS. However, recent studies, mainly employing the Lilly antagonist, LY382884, have identified several functions: For example, at mossy fibre synapses in the hippocampus these receptors function as facilitatory autoreceptors and are involved the induction of LTP. Kainate receptors also contribute to synaptic transmission at this synapse. Elsewhere kainate receptors can function as inhibitory autoreceptors and can regulate GABA transmission.

Whilst LY382884 is a very useful antagonist it has a relatively narrow selectivity for GluR5-containing kainate receptors *versus *AMPA receptors. David Jane and his colleagues in Bristol have therefore developed a series of highly potent and specific GluR5 antagonists, the most potent of which is ACET. This compound should be extremely useful in investigating the role of kainate receptors in physiological and pathological functions in the CNS, for example, in neurodegeneration and neuropathic pain.

• **Robin L Cooper (Department of Biology, University of Kentucky, Lexington, USA) – Effects of the serotonergic system on physiology, development, learning and behavior of drosophila melanogaster**

The serotonergic system in nervous tissue is known to play a vital role in development and behavior in simple to complex animal models. Using a simple model organism, Drosophila, the importance of serotonin (5-HT) circuitry in development and acute actions can be addressed. Also there are only four 5-HT receptors in the Drosophila genome, of which 5-HT2dro is known to be essential in the embryonic stages of development. Previously we have shown a physiological sensitivity of exogenous application of 5-HT on a sensory-CNS-motor circuit in semi-intact preparations of 3rd instar larvae. Now, using pharmacological manipulations and available receptor mutants for 5-HT2dro, we are studying the role of 5-HT in development, behavior and physiology of 3rd instar larvae. Para-chlorophenylalanine (p-CPA), is a blocker of 5-HT biosynthesis pathway and 3,4-Methylenedioxymethamphetamine (MDMA, Ecstasy), is a common drug of abuse in humans, which is known to compel mammalian serotonergic neurons to release 5-HT. When fed these compounds from 1st to 3rd instar a slowing of the growth occurred in a dose dependent manner. The rate of body wall and mouth hook movements were reduced in p-CPA and MDMA fed larvae. HPLC results showed lower amounts of 5-HT in larval brains for p-CPA but not MDMA fed larvae. An increase in sensitivity of sensory-CNS-motor circuit to 5-HT in drug fed larvae appears to be due an up regulation of 5-HT receptors. The antisense line for 5-HT2dro receptor also produces a delay in larval development. Preliminary data shows an impaired associative gustatory and olfactory learning behaviors in 3rd instar larvae with lower 5-HT or reduced expression of the 5-HT2dro receptor.

• **Jens R Coorssen (Department of Physiology and Biophysics, University of Calgary, Calgary, Canada) -The role of cholesterol in synaptic release**

Fast, Ca^2+ ^-triggered membrane merger defines regulated exocytosis. In native secretory vesicles, cholesterol (CHOL) functions in the fundamental fusion mechanism, and CHOL/sphingomyelin – enriched microdomains define the efficiency (Ca^2+ ^sensitivity and kinetics) of fusion. The role of CHOL in the fusion mechanism is mimicked by structurally dissimilar lipidic membrane components having spontaneous negative curvature (/NC/) = CHOL, and correlates quantitatively with the/NC/each contributes to the membrane (e.g. a-tocopherol and dioleoylphosphatidylethanolamine). Unable to substitute for CHOL in rafts, these lipids do not rescue fusion efficiency. Lipids of spontaneous/NC/< CHOL (e.g. dioleoylphosphatidic acid), do not support fusion. We have also identified com-parable molecular dependencies and relationships at the synapse, suggesting a conserved role for CHOL and the/NC/contributed. This quantitative relationship between/NC/and fusion appears most consistent with the stalk-pore model, demonstrating that/NC/itself is an essential component of the fundamental native fusion mechanism. The data also suggest that different fusion sites, vesicles, or secretory cells can use other lipidic components, in addition to sterols, to provide optimal local/NC/and even to modulate the fusion process.

• **Alaa El-Husseini (Department of Psychiatry, University of British Columbia, Vancouver, Canada) – Elaboration of dendritic filopoidia is not a rate-limiting step for production of stable axonal-dendritic contacts.**

Dendritic filopodia are thought to play an active role in synaptogenesis and serve as precursors to spine synapses. However, this hypothesis is largely based on a temporal correlation between the onset of filopodia elaboration and synaptogenesis. We have previously demonstrated that the palmitoylated protein motifs of GAP-43 and paralemmin are sufficient to increase the number of filopodia and dendritic branches in neurons. Here we examined whether filopodia induced by these motifs, as well as those induced by cdc42 lead to the formation of stable synaptic contacts and the development of dendritic spines. Our analysis shows that expression of these filopodia inducing motifs (FIMs) or the constitutively active form of cdc-42 enhances filopodia motility, but reduces the probability of forming a stable axon-dendrite contact. Conversely, expression of neuroligin-1 a synapse inducing cell adhesion molecule, resulted in a decrease in filopodia motility, an increase in the number of stable axonal contacts, and the recruitment of synaptophysin positive transport packets. Postsynaptic scaffolding proteins such as Shank-1 that induce the maturation of spine synapses reduced filopodia number, but increased the rate at which filopodia transformed into spines. By following individual dendrites over a 2-day period we determined that relatively few sites with filopodia are replaced by spine synapses (~3%). These results suggest that high levels of filopodia elaboration and motility may not necessarily be a rate-limiting step for synapse formation, and that factors that control filopodia-process dynamics may participate in synapse formation by rapid stabilization of the initial contact between dendritic filopodia and axons.

• **Vasco Galhardo (Institute for Molecular and Cell Biology, University of Porto, Porto, Portugal) – Impairment in prefrontal-based emotional decision-making in rat models of chronic pain**

Chronic pain is known to cause several cognitive deficits in human subjects. Among these deficits is the incapability of performing correctly in decision-making tasks that have a risk component, such as rewards of variable value. This cognitive impairment is known to occur after amygdalar or orbitofrontal lesions, where individuals are incapable of long-term planning and take high-risk decisions even if they lead to overall losses. It was recently shown that chronic pain patients also present this pattern of impaired decision-making (Apkarian et al, Pain, 108:129, 2004). However, no studies in chronic pain animal models have addressed poor performance in frontal-based cognitive tasks. For this reason we developed a novel behavioural task based on repetitive reward-based simple decisions, and studied its performance by both control, frontal-lesioned, and chronic pain animals (n = 6 per group). The task consisted on consecutive trials in which a rat entered an operant chamber and had to choose between two levers to recover a food reward. After each trial, the animal was removed to a contiguous chamber where he waited for a sound signal to begin a new trial. During the 15 days of the training phase both levers gave equal pseudo-random rewards: one food pellet in 8 of 10 presses, and no reward in the other two – low risk. In the trial probe one of the levers was modified to give 3 food pellets, but only in 3 of 10 visits – high risk. The pattern of 120 consecutive choices was used to calculate the lever-choice index (low risk minus high risk, divided by number of completed trials). In the first 60 trials all the animals (controls, lesioned and monoarthritic) reverently choose the large reward lever, but control animals reversed the pattern of choice in the second half of the session. When analyzing the last 30 entries, controls had a choice index of +0.42 ± 0.17, while monoarthritic rats had -0.48 ± 0.14, neuropathic rat had -0.53 ± 0.21 and frontal-lesioned animals -0.58 ± 0.31. We have shown for the first time that chronic pain induces complex changes in the cognitive neural processes that handle immediate decision-making in the rat (Support: FCT-POCI/55811/2004).

• **Wen-Biao Gan (Skirball Institute, New York University School of Medicine, New York, USA) – Dendritic Spine Stability And Its Modification By Experience**

The nervous system requires not only synaptic plasticity for learning but also stability for long-term information storage. To study the degree of synaptic structural plasticity in intact animals, we developed a transcranial two-photon imaging technique to follow individual postsynaptic dendritic spines over time in transgenic mice over-expressing Yellow Fluorescent Protein. Using this technique, we found that in young adolescent mice (1-month-old), 13–20% of spines were eliminated and 5–8% were formed over 2 weeks in visual, barrel, motor and frontal cortices, indicating a cortical-wide loss of spines during this developmental period. In adult mice (4–6 months old), 3–5% of spines were eliminated and formed over 2–4 weeks in various cortical regions. When imaged over 19 months, only 26% of adult spines were eliminated and 19% were formed in barrel cortex. Thus, after a concurrent reduction in the number of spines in the diverse regions of young adolescent cortex, spines become remarkably stable and a majority of them can last throughout life.

To determine how spine dynamics are modified by experience, we examine the effect of long-term sensory deprivation via whisker trimming on dendritic spines in the barrel cortex. During young adolescence when a substantial net loss of spines occurs, we found that whisker trimming preferentially reduces the rate of on-going spine elimination than spine formation. This effect of deprivation diminishes as animals mature but still persists in adulthood. In addition, restoring sensory experience following adolescent deprivation accelerates spine elimination but has no significant effect on spine formation. The rate of spine elimination also decreases after chronic blockade of NMDA receptors with the antagonist MK801 and accelerates after drug withdrawal. These studies underscore the important role of sensory experience in spine elimination over the majority of an animal's life span, particularly during adolescence.

• **Jianguo Gu (Department of Oral and Maxillofacial Surgery, University of Florida, Gainesville, USA) – TRPM8 and cold allodynia**

Peripheral nerve injury often results in neuropathic pain manifested with both mechanical and thermal allodynia. Thermal allodynia of neuropathic pain conditions includes cold- and heat allodynia. While TRPV1 is found to be involved in heat allodynia, molecular mechanisms of cold allodynia remain unclear. Recently, transient receptor potential channel M8 (TRPM8 receptor) is found to be a cold- and menthol-sensing receptors expressed on a subpopulation of primary afferent fibers. Here we report the upregulation of TRPM8 expression on nociceptive-like afferent neurons following chronic constrictive nerve injury (CCI) rats that manifested with cold allodynia. We found not only the number of TRPM8-expressing neurons was increased, but also the responsiveness to cold and menthol became enhanced in these afferent neurons following CCI. These results suggest TRPM8 upregulation is associated with cold allodynia and may be an underlying mechanism of cold allodynia

• **Kazuhide Inoue (Department of Pharmaceutical Health Care and Sciences, Kyushu University, Kyushu, Japan) – P2X4: mechanisms of over expression in neuropathic pain state**

There is abundant evidence that extracellular ATP and other nucleotides have an important role in pain signaling at both the periphery and in the CNS. Recent findings suggest that endogenous ATP and its receptor system might be involved in neuropathic pain. Neuropathic pain is often a consequence of nerve injury through surgery, bone compression, diabetes or infection. This type of pain can be so severe that even light touching can be intensely painful; unfortunately, this state is generally resistant to currently available treatments. We recently reported that the expression of P2X4 receptors in the spinal cord is enhanced in spinal microglia after peripheral nerve injury, and blocking pharmacologically and suppressing molecularly P2X4 receptors produce a reduction of the neuropathic pain behaviour (Nature 424,778–783, 2003), and that brain-derived neurotrophic factor (BDNF) released from microglia by the stimulation of P2X4 causes the depolarizing shift in reversal potential of anion in LI neurons of rats with nerve injury (Nature, 438, 1017–1021, 2005), resulting in causing neuropathic pain. Understanding the key roles of these ATP receptors may lead to new strategies for controlling the pain.

• **John Isaac (NINDS, NIH, Bethesda, USA) – Rapid, Activity-Dependent Plasticity in Timing Precision in Neonatal Barrel Cortex**

During development neuronal networks acquire the ability to precisely time events. This is a critical developmental step since precise timing is required for information processing and plasticity in the adult brain. Despite this it is not known what process drives this maturation in timing. I will present recent work from my laboratory showing that long-term potentiation (LTP) induced at thalamocortical synapses in neonatal layer IV barrel cortex produces a rapid and dramatic improvement in input and output timing precision. LTP reduces the latency and variability of synaptically-evoked action potentials and reduces co-incidence detection for synaptic input. In contrast, LTP has only a small and variable effect on synaptic efficacy. This improvement in timing occurs during development, suggesting this process occurs in vivo in the developing barrel cortex. Thus, rather than increasing synaptic efficacy, the primary role of this form of neonatal LTP is to enable neurons to precisely time events.

• **Koichi Iwata (Department of Physiology, Nihon University, Tokyo, Japan) – Anterior cingulate cortex and pain -its morphological feature and functional properties**

It is well known that the anterior cingulate cortex (ACC) has a variety of functions related to pain including pain perception. Many ACC neurons respond to noxious and non-noxious stimulation of the body. Most of these neurons have a large receptive field and increase their firing frequency as stimulus intensity increases. ACC nociceptive neurons have very specific morphological features, such as a small soma and a large number of spines on the dendritic trees, and axon collaterals spreading over a wide area of the ACC. In a retrograde trans-synaptic tracing study, we found that ACC neurons receive predominantly A-delta afferents inputs. We also analyzed the responses of ACC nociceptive neurons in awake behaving monkeys. A small number of ACC neurons modulated their activity during noxious heating of the facial skin. The neuronal activity was significantly higher when monkeys escaped from a noxious heat stimulus than when the monkeys detected a small change in temperature (T2) above a larger initial shift (T1). No relationship between firing frequency and detection latency of the T2 stimulation was observed. These findings suggest that ACC nociceptive neurons are involved in attention to pain and escape from pain, but not in the sensory-discriminative aspect of pain.

• **Zhengping Jia (Department of Physiology, University of Toronto, Toronto, Canada) – Molecular regulation of spine properties and synaptic plasticity**

The dendritic spine is the major postsynaptic site of excitatory synapse and its changes are linked to synaptic plasticity, memory formation and various forms of mental and neurological disorders. However, the molecular mechanisms that govern spine development and regulation are poorly defined. We take genetic approaches in mice to identify and characterize the molecular signaling processes involved in the regulation of spine formation, spine morphology, and spine and synaptic plasticity. Specifically, we are interested in the signal transduction pathways stimulated by the Rho family small GTPases, key mediators of actin dynamics in response to various external stimuli. Our objective is to define the in vivo function and synaptic regulation of Rho signaling in the context of spine properties, hippocampal long-term potentiation and fear memory formation. The specific roles and the underlying mechanisms of various components required for normal Rho signaling will be discussed.

• **Bong-Kiun Kaang (Department of Biological Sciences, Seoul National University, Seoul, Korea) – Role of a novel nucleolar protein ApLLP in synaptic plasticity and memory in Aplysia**

In Aplysia long-term synaptic plasticity is induced by serotonin (5-HT) or neural activity, and requires gene expression. Here, we demonstrate that ApLLP, a novel nucleolus protein is critically involved in both long-term facilitation (LTF) and behavioral sensitization. Membrane depolarization induced ApLLP expression, which activated ApC/EBP expression through a direct binding to CRE. LTF was produced by a single pulse of 5-HT 30 min after the membrane depolarization. This LTF was blocked when either ApLLP or ApC/EBP were blocked by specific antibodies. In contrast, ApLLP overexpression induced LTF in response to a single 5-HT treatment. Simultaneously, a siphon noxious stimulus (SNS) to intact Aplysia induced ApLLP and ApC/EBP expression, and single tail shock 30 min after SNS transformed short-term sensitization to long-term sensitization of siphon withdrawal reflex. These results suggest that ApLLP is an activity-dependent transcriptional activator that switches short-term facilitation to long-term facilitation

• **Mikito Kawamata (Department of Anesthesiology, Sapporo Medical University School of Medicine, Sapporo, Japan) – Genetic variation in response properties of spinal dorsal horn neurons and rostral ventromedial medulla neurons in different mouse strains**

Although various methods of analgesia are currently used for persistent pain such as inflammatory pain and neuropathic pain, optimal pain therapy has still not been established. This may be at least in part related to variability of perceived pain among patients. Recent behavioral studies have shown that nociception in the mouse is heritable, which may reflect variable sensitivity to tissue injury-induced pain in humans (Mogil et al., 1996). Noxious information is transmitted through fine myelinated Aδ and unmyelinated C afferents from the periphery to the superficial dorsal horn (SDH), especially to the substantia gelatinosa (SG, lamina II of Rexed) (Light and Perl, 1979). This sensory information is modified and integrated in the SG and consequently regulates the outputs of projection neurons located in lamina I and laminae V-VI (Cervero and Iggo, 1980; Eckert et al., 2003). In addition, the descending inhibitory influences from supraspinal structures, including the rostral ventromedial medulla (RVM), on SDH neurons are known to be modified under certain pathological conditions (Basbaum, 1973; Dubuisson and Wall, 1980; Laird and Cervero, 1990; Sandkuhler et al., 1995; Wall et al., 1999).

Thus, nociceptive network circuits in the central nervous system, including the SDH and RVM, may play an important role in different pain sensitivity in individuals. Our hypothesis is that SDH neurons and RVM neurons in different mouse strains may show different response properties following tissue injury and different sensitivity to analgesics depending on different genetic background. In order to prove this hypothesis, *in vivo *extracellular recordings and *in vivo *whole-cell patch-clamp recordings were made from SDH neurons located in deep laminae (laminae V-VI) and from superficial SDH neurons located in lamina II, respectively, in different strains of mice (A/J, C57BL/6J, and CBA/J strains) before and after tissue injury induced by surgical incision and formalin injection according to previously described methods (Furue et al., 1999; Kawamata et al, 2005). In a separate study, single neuronal activity was isolated from different types of RVM neurons such as ON cells, OFF cells and NEURTRAL cells, and response properties of these neurons were determined before and after intraventricular injection of DAMGO or surgical injury.

The results have shown that different mouse strains have different sensitivities to postoperative pain and formalin-induced pain, reflecting different characteristics of SDH neurons in the strains following surgical incision and application of formalin. Responses of RVM neurons were also different in different mouse strains following surgical injury and that different mouse strains have different sensitivities to morphine application. The results suggest that pain intensity and pain mechanisms depend at least in part on genetic background of the individual. Furthermore, mechanisms of pain seen in a clinical setting may thus differ in individuals depending on the response properties of SDH neurons and RVM neurons.

• **Satoshi Kida (Department of Agricultural Chemistry, Tokyo University of Agriculture, Tokyo, Japan) – Mechanism of interaction between reconsolidation and extinction of contextual fear memory**

Retrieval of conditioned fear memory initiates two potentially dissociable but opposite processes; reconsolidation and extinction. Reconsolidation acts to stabilize, whereas extinction tends to weaken the expression of the original fear memory. To understand the mechanisms for the regulation of memory stability after the retrieval, we have investigated the relationship between reconsolidation and extinction using contextual fear conditioning, associative learning between context (conditioned stimulus; CS) and fear (unconditioned stimulus; US). We first examined effects of duration of re-exposure to CS on memory reconsolidation and extinction. Protein synthesis inhibition following short re-exposure (3 min) to CS disrupted the contextual fear memory, indicating short re-exposure induces memory reconsolidation. In contrast, protein synthesis inhibition following long-re-exposure (30 min) blocked memory extinction. Importantly, in extinction phases, contextual fear memory was intact even though protein synthesis was inhibited. Therefore, these observations suggested the interaction between memory reconsolidation and extinction phases. Indeed, memory extinction seems to be associated with regulation of fear memory stability after retrieval.

To further understand how extinction phase interacts with reconsolidation phase, we assume that molecules functioning on one phase should also function on another phase if interaction between reconsolidation and extinction phases is observed at the molecular level. Therefore, we compared molecular signatures of these processes using pharmacology and mouse genetics. Pharmacological experiments using antagonists for cannabinoid receptor 1 (CB1) and L-type voltage-gated calcium channels (LVGCCs), that play essential roles in memory extinction, indicated that both CB1 and LVGCCs are required for memory extinction but not consolidation and reconsolidation. More importantly, double injection of anisomycin and antagonists for either CB1 or LVGCCs prevents the disruption of the original memory by protein synthesis. These results suggest that CB1 and LVGCCs are required for not only memory extinction but also the destabilization of reactivated memory. We are now trying similar experiments using conditional CREB mutant mice.

In addition, to compare the brain regions associated with reconsolidation and extinction, we analyzed brain regions showing increase in CREB activity in reconsolidation and extinction phases by immunocytochemistry. We observed increase in phosphorylated CREB at serine 133 in amygdala and hippocampus following short re-exposure to CS inducing memory reconsolidation and in amygdala and prefrontal cortex following long re-exposure to CS inducing memory extinction. These observations suggest that acquisition of memory extinction prevent the activation of hippocampus, resulting in preservation of contextual fear memory.

Taken together, these our findings indicate the interaction between memory extinction and regulation of memory stability at the molecular, anatomical and behavioral level. Further understanding the mechanisms of this interaction might make more clearly understand the significance of memory reconsolidation.

• **Eric Klann (Department of Molecular Physiology and Biophysics, Baylor College of Medicine, Houston, USA) – Translational Control During Hippocampal Synaptic Plasticity and Memory**

Altered gene expression is a hallmark of long-lasting synaptic plasticity and long-term memory. Regulation of local protein translation permits synapses to control synaptic efficacy independently of mRNA synthesis in the cell body. Recent studies, including several from this laboratory, have identified biochemical signaling cascades that couple neurotransmitter and neurotrophin receptors to the translation regulatory machinery in translation-dependent forms of synaptic plasticity and memory. In this presentation, these translation regulatory mechanisms and the signaling pathways that govern the expression of various forms of translation-dependent synaptic plasticity and memory will be discussed. In addition, synaptic plasticity and memory deficits in genetically engineered mice that lack specific translation factors and translation regulatory proteins will be discussed. These studies have revealed interesting links among the biochemical activities of translation factors, synaptic plasticity, and memory that are likely to be important for other forms of plasticity and behavior, such as those that underlie pain and drug addiction.

• **Tatsuro Kohno (Division of Anesthesiology, Niigata University, Niigata, Japan) – Different actions of opioid and cannabinoid receptor agonists in neuropathic pain**

Peripheral nerve injury causes neuropathic pain, which is characterized by hyperalgesia and allodynia to mechanical and thermal stimuli. Neuropathic pain has traditionally been considered opioid-resistant to intrathecal opioids; however, the efficacy of opioid in treating neuropathic pain is controversial. In contrast, increasing evidence indicates that cannabinoids are effective in alleviating neuropathic pain. We evaluated the effect of opioids and cannabinoids in two independent partial peripheral nerve injury models, the spared nerve injury (SNI) and the spinal nerve ligation (SNL) models. In both the SNI and SNL rat peripheral neuropathic pain models the presynaptic inhibitory effect of the μ opioid receptor (MOR) agonist (DAMGO) on primary afferent-evoked excitatory postsynaptic currents (EPSCs) and miniature EPSCs in superficial dorsal horn neurons is substantially reduced, but only in those spinal cord segments innervated by injured primary afferents. The two nerve injury models also reduce the postsynaptic potassium channel opening action of DAMGO on lamina II spinal cord neurons, but again only in segments receiving injured afferent input. The inhibitory action of DAMGO on ERK (extracellular signal-regulated kinase) activation in dorsal horn neurons is also reduced in affected segments following nerve injury. MOR expression decreases substantially in injured dorsal root ganglion neurons (DRG), while intact neighboring DRGs are unaffected. In contrast to MOR agonist, the selective CB1 receptor agonist (ACEA) still suppressed C-fiber-induced ERK activation in dorsal horn neurons in injured spinal cord segments from SNL rats. These studies suggest that opioids may reduce sensitivity in those patients whose pain is generated mainly from injured nociceptor discharge. However, opioid may still be able to suppress neuropathic pain via acting on intact primary afferents or via supraspinal mechanisms. Because the efficacy of cannabinoid in suppressing C-fiber-induced ERK expression fully remains in the injured spinal segments after nerve ligation, our results support an undiminished potency of cannabinoid in attenuating neuropathic pain. Our data also suggest that there might be different regulatory mechanisms of opioids and cannabinoids for neuropathic pain.

• **Min Li (Department of Neuroscience and High Throughput Biology Center, Johns Hopkins University, Baltimore, USA) – Chemical regulation of membrane excitability**

Biological phenomena – ranging from neuronal action potential, to rhythmic cardiac contraction, to sensory transduction, to hormone secretion – are ultimately controlled by one class of proteins: the ion channels. Changes of ion channel activities by genetic mutations or by drugs are causes for human diseases and basis for therapeutics. Potassium channels are critical to a variety of biological processes and represent a very large class of ion channel proteins permeable to potassium ions. Of the more than 400 ion channel genes in the human genome, at least 167 are annotated potassium channels.

The regulation and biogenesis of potassium channels are important processes essential to the understanding of their physiological roles. Recent evidence indicates that cardiotoxicity of many human drugs for other intended targets is caused by inhibition of a subset of potassium channels through different mechanisms. These drugs are both chemically stable and economically available. Therefore, they represent useful chemical probes to investigate potassium channel regulation both at the molecular level and at the cell biological level. Using a combination of high throughput chemical biology approaches and detailed biochemical and electrophysiological analyses, we have screened and identified a number of regulatory compounds with unique mechanisms of action in regulating potassium channels.

• **Xiao-Jiang Li (Department of Human Genetics, Emory University, Atlanta, USA) – Synaptic toxicity of Huntington disease protein**

Huntington's disease (HD) is characterized by the selective loss of striatal projection neurons. In early stages of HD, neurodegeneration preferentially occurs in the lateral globus pallidus (LGP) and substantia nigra (SN), two regions where the axons of striatal neurons terminate. The unique neuronal structure, which is characterized by numerous neuronal processes that interact with each other at their terminals, may confer the preferential vulnerability to expanded polyQ proteins. In HD mice that precisely and genetically mimic the expression of full-length mutant huntingtin (htt) in HD patients, we found that degraded N-terminal fragments of htt preferentially forms aggregates in striatal neurons that are most affected in HD. More importantly, neuropil aggregates form preferentially in the processes of striatal neurons. In HD transgenic mice that express N-terminal mutant htt, the progressive formation of these neuropil aggregates correlates with disease progression. We also observed degenerated axons in which htt aggregates were associated with dark, swollen organelles that resemble degenerated mitochondria. These findings suggest that the early neuropathology of HD originates from axonal dysfunction and degeneration associated with htt neuropil aggregates.

• **John F MacDonald (Department of Physiology, University of Toronto, Toronto, Canada) – Inhibitory Regulation of the Src Hub and LTP in CA1 Hippocampal Neurons**

The induction of long-term potentiation (LTP) at CA1 synapses of the hippocampus requires an influx of Ca^2+ ^via N-methyl-d-aspartate receptors (NMDARs). High frequency stimulation depolarizes CA1 neurons, relieving the voltage-dependent block of NMDARs by Mg^2+^, permitting the entry of Ca^2+ ^that is critical for this induction. Thus, NMDARs serve as co-incident detectors of the LTP-inducing afferent input to CA1 neurons. Enhanced activation of the non-receptor tyrosine kinase Src is also required for this co-incidence function; and, Src is the convergent target of a variety of G-protein coupled receptors (GPCRs) of the Gα q family (e.g. LPA, muscarinic, mGluR5 and PACAP receptors). These GPCRs stimulate a Src-dependent upregulation of NMDARs via a sequential activation of PKC and the non-receptor tyrosine kinase, Pyk2 which is also required for induction of LTP. Src therefore acts as a hub for the regulation of the induction of LTP at CA1 synapses.

Signaling pathways which inhibit Src, and thereby inhibit the induction of LTP, have not been extensively studied. We have previously shown that platelet-derived growth factor receptors (PDGFRβ) inhibit NMDARs in CA1 neurons by a PKA-dependent but Src-permissive mechanism. For example, in inside-out patches from cultured hippocampal neurons PKA fails to inhibit NMDAR channel activity unless it is first enhanced with a Src-activator peptide. Furthermore, we show that in hippocampal slices PDGFBB (receptor ligand) inhibits the induction of LTP. The initial step in this pathway requires tyrosine phosphorylation of tyrosine 1021 of the PDGFR which forms a SH2 docking site for PLCγ. PLCγ interacts with another non-receptor kinase, Abelson kinase (Abl) which among other activities regulates PDGFR activity via a biochemical feedback. In recordings from single isolated CA1 pyramidal neurons we show that intracellular applications of Abl kinase strongly inhibit currents evoked by applications of NMDA. This inhibition is reversibly blocked by extracellular applications the PDGFR antagonist gleevec demonstrating the dependency of this response on PDGFR activity. How PDGFR, PLCγ and Abl kinase activity translates into inhibition of NMDARs is not fully understood and is currently under investigation.

• **Karim Nader (Department of Psychology, McGill University, Montreal, Canada) – Identifying the neural mechanisms by which boundary conditions inhibit reconsolidation from occurring.**

Although memory reconsolidation has been demonstrated in various learning tasks and animal models suggesting it is a fundamental process, reports of boundary conditions imply that reconsolidation is not ubiquitous. These boundary conditions, however, remain poorly defined at the behavioral, systems and molecular levels. Attempting to ameliorate this situation, we characterized reconsolidation of strong memories across all three levels of analysis. At the behavioral level we demonstrated that this boundary condition is transient, as infusions of anisomycin into lateral and basal amygdala in rats did not impair reconsolidation of overtrained auditory fear memories after 2 or 7 days, but did so after 30 or 60 days after training. At the systems level we showed that the hippocampus imposes the boundary condition on the amygdala, as the overtrained memory underwent reconsolidation 2 days after training in animals with pretraining dorsal hippocampus lesions. At the molecular level we demonstrated that the degree of expression of NR2B-containing NMDA receptors in the amygdala modulates reconsolidation of overtrained fear memories, as these receptors, which we previously have identified as being essential for the transformation of a consolidated memory back to a labile state, were down-regulated 2, but not 60 days after overtraining; furthermore, animals with pre-training hippocampus lesions, that did not exhibit the overtraining boundary conditions two days after training, had normal level of expression of NR2B subunits at that time-point. These findings make three conceptual advances in our understanding of reconsolidation: first, boundary conditions can be transient, second, boundary conditions can be imposed by other brain systems, and third, a mechanism mediating the manifestation of boundary conditions is down-regulation of the receptors that are critical for inducing reconsolidation.

• **Peter V Nguyen (Department of Physiology, University of Alberta, Edmonton, Canada) – Beta-Adrenergic Receptors Recruit ERK and mTOR to Promote Translation-Dependent Synaptic Plasticity**

A key question in neuroscience research is: How does activation of neuromodulatory receptors initiate protein synthesis during long-term synaptic plasticity? Activation of beta-adrenergic receptors can enhance long-term memory and modulate long-term synaptic plasticity in the mammalian hippocampus. Protein synthesis is required for the persistence of long-term potentiation (LTP) and for the consolidation of long-term memory. However, the intracellular signaling cascades that couple beta-adrenergic receptors to translation initiation and subsequent protein synthesis are unidentified. We used electrophysiological recordings in area CA1 of mouse hippocampal slices to investigate the recruitment of signaling cascades necessary for beta-adrenergic LTP. We found that maintenance of this LTP requires the extracellular signal-regulated kinase (ERK) and mammalian target of rapamycin (mTOR) pathways, but not cAMP-dependent protein kinase (PKA). Consistent with these findings, treatment of hippocampal slices with isoproterenol, a beta-adrenergic agonist, increases phosphorylation of eukaryotic initiation factor 4E (eIF4E), the eIF4E kinase Mnk1, and the translation repressor, 4E-BP2. These translational regulators can be phosphorylated in an ERK- and mTOR-dependent manner. Moreover, activation of beta-adrenergic receptors eliminates deficits in late-LTP seen in transgenic mice that express reduced hippocampal PKA activity. Our results identify specific intracellular signaling pathways that link beta-adrenergic receptor activation at the membrane to translation initiation within the cytosol. More importantly, our data reveal a molecular mechanism for neuromodulatory control of protein synthesis during LTP, a process that is required for the formation of long-lasting memories. [Funded by Alberta Heritage Fdn. for Med. Res. and CIHR, NIH, NIMH, and the Fragile X Research Fdn].

• **Uhtaek Oh (Sensory Research Center, Seoul National University, Seoul, Korea) – TRPV1 and its Role for Inflammatory Pain**

Capsaicin (CAP) is a pungent ingredient in hot peppers. CAP has a unique action on the pain sensory system. CAP causes a pain when applied to the skin. The hyperalgesic action of CAP is mediated by the excitation of sensory neurons. CAP is known to activate ion channels that allow cation influxes, thus, depolarizing sensory neurons. CAP-activated ion channel along with its channel property was identified. The channel is a ligand-gated channel and permeable to various cations. The gene encoding for the CAP sensitive current was cloned and dubbed as VR1 (vanilloid receptor 1). Primary structure of VR1 shows that VR1 belongs to transient receptor potential (TRP) channel family, having 6 transmembrane domains with two long cytosolic amino acid sequences in each N- or C- terminus. According to the recently classified nomenclature, VR1 is now classified as TRPV1. Mice deficient of TRPV1 lacks thermal pain induced by inflammation. Thus, TRPV1 is most likely involved in the mediation of inflammatory pain. In the present symposium, I would like to introduce our research on TRV1, most notably, presenting evidence for the involvement of TRPV1 in mediating inflammatory pain signaling pathways.

The presence of TRPV1 receptor and its apparent role in pain suggests endogenous activator. Thus, endogenous activators of TRPV1 were searched. In our previous report, the hyperalgesic neural response such as c-fos expression in the dorsal horn of the spinal cord induced by inflammation is blocked by capsazepine, a CAP receptor blocker, suggesting that an endogenous capsaicin-like substance is produced and causes hyperalgesia by opening capsaicin-activated channels. Because ligands bind from the intracellular side of the channel, the endogenous ligands would likely be produced in the cell. We initially tested many intracellular messengers on the CAP channel to determine whether they activate the channel. We found that products of lipoxygenases (LO) are capable of activating the channel. Interestingly, products of LOs are implicated in mediating inflammatory nociception because various LO products are produced during inflammation and cause hyperalgesia when injected intradermally. In addition, products of LOs often function as intracellular messengers in neurons. Among their actions, products of LOs act directly on K+ channels in Aplysia sensory neurons (Piomellile et al., 1987) and mammalian cardiac muscle cells.

In the present seminar, we present evidence that products of LOs directly activate the CAP receptor in isolated membrane patches of sensory neurons. When applied to the bath of inside-out patches, 12-hydroperoxytetraenoic acid (12-HPETE) activates single-channel currents that were sensitive to capsazepine in isolated membrane patches. The IV curve of single-channel currents activated by 12-HPETE is outwardly-rectifying and identical to that obtained by CAP. The amplitude of single-channel currents activated by both 12-HPETE and CAP are not different. Theses results indicate that the channel currents activated by 12-HPETE are identical to those activated by CAP. The channels activated by 12-HPETE are permeable to various cations. LO products also activate TRPV1, the cloned CAP receptor, expressed HEK293 cells. Products of LOs other than 12-HPETE also activated the CAP channels. Among them, 12- and 15-HPETE, 5- and 15-(S)-hydroxyeicosatetraenoic acids, and leukotriene B4 possess the highest potency. Dose-response relationships reveal that the potencies of 12-HPETE, 15-HPETE, leukotrien B4, and 5-HETE are 8.0, 8.7, 9.2, and 11.7 μM, respectively, showing much lower potency than CAP. Anandamide, the endogenous ligand for cannabinoid receptors also activates the channel with half-maximal dose of 11.7 μM. Because prostaglandins (PGs) are known to be related to pain, various PGs are applied to the CAP receptors. PGs, however, fail to activate the channel. Other saturated or unsaturated fatty acids are also tested for its activation of CAP channels. They all fail to activate the channels.

Results of our study indicate that CAP and various eicosanoids act on the capsaicin receptor, suggesting a structural similarity between CAP and eicosanoids. Thus, structures of eicosanoids and CAP in the energy-minimized state are superimposed to compare three-dimensional structures. Three-dimensional structures of 12-(S)-HPETE, 15-(S)-HPETE, 5-(S)-HETE, and LTB4 are compared with that of CAP. Interestingly, CAP in the energy-minimized state fits well to the S-shaped 12-HPETE. In particular, the phenolic hydroxide and amide moieties in CAP overlap precisely with the carboxylic acid and hydroperoxide moieties in 12-HPETE, respectively. The two key regions in CAP or 12-(S)-HPETE are known to have dipolar property that allows hydrogen bond interactions with the CAP receptor. In addition, the aliphatic chain region of the 12-(S)-HPETE fits well with the alkyl chain of CAP. In contrast, 15-HPETE, 5-HETE and LTB4, shared less structural similarity with CAP.

Because products of LO activate the channel, it seems obvious to ask what stimulates the LO/TRPV1 pathway in order to cause pain. Although bradykinin (BK) is a powerful pain causing inflammatory mediator, but its activation mechanism of sensory neurons is not known. Because BK releases arachidonic acid, a key substrate for LO in sensory neurons, we hypothesized that BK activates TRPV1 via the PLA2/LO pathway. In order to prove the hypothesis, we performed electrophysiological experiments, Ca2+-imaging, and chemical analysis of LO products. As a result, we observed that BK-evoked whole-cell currents recorded from sensory neurons were significantly reduced by capsazepine (CZP), a capsaicin receptor antagonist. In the skin nerve preparation, CZP and quinacrine, a PLA2 inhibitor, and NDGA, a LO inhibitor reduced BK-induced excitation of sensory nerves. In addition, quinacrine, NDGA and CZP blocked BK-induced Ca2+-influx. To examine if sensory neurons can, in fact, release the lipid products of LO by BK, we used HPLC-coupled with radioisotope to detect the lipid products. As results, we confirmed that 12-HETE, an immediate downstream metabolite of 12-HPETE was indeed released from sensory neurons after the BK application.

In addition, we also present unequivocal evidence that histamine, another inflammatory mediator, also uses the PLA2/LO/TRPV1 pathway for excitation of sensory neurons. Application of histamine caused influx of Ca2+, which was blocked by co-application of capsazepine or Ca2+-free condition. The Ca2+-influx induced by histamine was blocked by application of capsazepine. Likewise, the Ca2+-influx induced by histamine was also blocked by treatment of NDGA or quinacrine. Thus, these results now suggest that histamine activates TRPV1 by stimulation of PLA2 and LO. Because histamine is a major pruritogenic (itch causing) substance, identification of the histamine signaling pathway is much helpful to developing anti-pruritogenic substance to cure itch sensation in atopic dermatitis patients.

This study demonstrates that bradykinin and histamine excite sensory nerve endings by activating TRPV1 via production of 12-LO metabolites of arachidonic acid by activated PLA2. This finding identifies a mechanism that might be targeted in the development of new therapeutic strategies for the treatment of inflammatory pain or itch.

• **Ke Ren (Department of Biomedical Sciences, University of Maryland, Baltimore, USA) – Neuronal/glial cell interactions in CNS plasticity and persistent pain**

Nerve signals arising from sites of tissue injury lead to long-term changes in the central nervous system (CNS) referred to as central sensitization. Ample evidence indicates that central sensitization underlies mechanisms of persistent pain after injury. The emerging literature strongly implicates a role of neuronal/glial cell interaction in central sensitization and hyperalgesia. Through still unknown mechanisms, glia can be activated after injury and release chemical mediators such as inflammatory cytokines that modulate neuronal activity and synaptic strength. Such glia-cytokine-neuron interactions may be critical in the chronic pain process. We tested this hypothesis in a rat model of synaptic plasticity and persistent pain. Tissue injury was produced by injecting complete Freund's adjuvant (CFA), an inflammatory agent, into the masseter muscle of the Sprague-Dawley rat. We first examined whether masseter inflammation induced glial activation in the spinal trigeminal complex (STC), the initial relay site for trigeminal nociceptive information. The results showed that masseter inflammation induced a selective and time-dependent increase in glial fibrillary acidic proteins (GFAP) levels, an indication of astroglial activation, in the STC. We next examined whether activation of glia by masseter inflammation is accompanied by an increase in inflammatory cytokine levels. Using Western blot and immunohistochemistry, an increase in IL-1beta in the STC was observed after masseter inflammation. The increase in IL-1beta was seen as early as 30 min after inflammation and lasted for about a week. Interestingly, the CFA-induced IL-1beta selectively colocalizes with GFAP, but not with NeuN, a neuronal marker, and CD11b, a marker of activated microglia. These results suggest that activated astrocytes are the source of IL-1beta release in the STC after masseter inflammation. To demonstrate the association of inflammation-induced cytokine release with glial activation, we tested the effect of propentofylline, a non-selective modulator of glia, on changes in GFAP and IL-1beta levels after masseter inflammation. Western blots showed that the propentofylline treatment blocked the increase in GFAP and IL-1beta after masseter CFA. We further showed that the increase in GFAP after masseter inflammation was blocked by local anesthesia of the injured site, suggesting its dependence on neuronal input. Interestingly, in a medullary slice preparation, substance P, a transmitter released from primary afferent terminals in the STC, induced an increase in GFAP and IL-1beta. These results are consistent with a role of neuronal signaling in triggering CNS glial activation. Finally, we tested the hypotheses that trigeminal glial activation and inflammatory cytokine release affect or facilitate neuronal plasticity through interactions with neuronal glutamate receptors. We administered IL-1receptor antagonist (ra) intrathecally via osmotic pumps at the level of the obex. The results showed that IL-1ra significantly attenuated behavioral hyperalgesia and blocked an increase in NMDA receptor phosphorylation after masseter inflammation. Our findings support a model of reciprocal neuron-glia interactions in the development of CNS plasticity and persistent pain. The model emphasizes activation of glia by injury-generated neuron input, concomitant cytokine release, and post-translational regulation of NMDA receptor sensitivity through IL-1receptor signaling. The outcome of these studies will help to identify novel targets and agents for clinical management of persistent pain. (Supported by NIH grants DE11964, DE15374, DA10275)

• **John C Roder (Department of Medical Genetics and Microbiology, University of Toronto, Toronto, Canada) – Forward and reverse genetic screens in the mouse for mutants impaired in learning and memory**

Learning and memory in the mouse is a quantitative trait and genes account for 71% of the variance between strains. Our goal here is to identify new genes that contribute to learning and memory. We will employ a forward genetic screen using a chemical mutagen (ENU). A total of 2500 ENU mice were pre-screened for normal development. Of these, 10 showed deficits in context-dependent fear conditioning (< 2 sd from the mean), but normal cue-dependent freezing. A smaller screen was done on 100 mice. 2 showed deficits in performance on the hidden platform but normal performance on the visible platform (control). All these presumed mutants showed low heritability and penetrance and could not be mapped to chromosomal positions. At this point we revised our screen.

A number of strains (n = 10) were compared in the water maze and the one showing optimal performance (129 S61SVE-v Tac) was chosen for ENU mutagenesis. In addition, we changed the screen to a much more difficult task and one that relied on a different sensory modality (sound) in trace conditioning.

Upon screening 450 mice in trace only, one was obtained that showed no freezing. This mutant showed robust inheritance and penetrance and we are in the process of fine mapping, positional cloning and sequencing of the antifreeze locus. Verification of candidate genes will be carried out by BAC rescue of the mutant phenotype with the wildtype gene. Alternatively, the creation of the same mutant phenotype in wildtype mice by mutating the wild-type locus in ES cells.

We will carry out extensive neurobehavioural assays to determine if the learning and memory deficits are restricted to the hippocampus or are found in other brain regions as well (i.e. pre-frontal cortex, cerebellum, nucleus accumbens, brain stem, amygdala, striatum). Learning and memory mutants will be tested for their ability to form cognitive spatial maps in the hippocampus in vivo. Neuroanatomical studies will assess if development perturbation underlie these deficits. Gene expression and proteomic studies will identify where the gene is expressed and the biochemical pathway underlying its action. Modifier screens will be carried out to elucidate exciting new genetic pathways. The mutant genes we identify will be models for human genetic diseases that involve impairments in learning and memory. In these cases, the mutant mice will provide test beds for pre-clinical tests of cognitive enhancers in patients. In addition, they will suggest new targets for drug development.

• **Michael W Salter (Department of Physiology, University of Toronto, Toronto, Canada) – Ins and outs of SRC regulation of NMDA receptors and synaptic plasticity**

Regulation of postsynaptic glutamate receptors is one of the principal mechanisms for producing alterations of synaptic efficacy in the CNS. A growing body of evidence indicates that at glutamatergic synapses NMDA receptors are upregulated by Src family tyrosine kinases which are opposed by the action of tyrosine phosphatases, one of which has been identified as STEP. Src itself is expressed nearly ubiquitously in higher organisms with the highest levels of expression found in the CNS. Src represents a point through which multiple signaling cascades from, for example G-protein-coupled receptors, Eph receptors and integrins, converge to upregulate NMDA receptor activity. The upregulation of NMDARs by activation of Src participates in the induction of long-term potentiation of synaptic transmission in the hippocampus and in the spinal cord dorsal horn. We have determined that Src is anchored within the NMDA receptor complex by the protein ND2. Recently, we have found that interfering with the ND2-Src interaction *in vivo *prevents behavioural pain hypersensitivity. Thus, multiple mechanisms control Src in the NMDA receptor complex and disrupting Src-mediated enhancement of NMDA receptor function affects pathological plasticity in the CNS.

• **Weihong Song (Department of Psychiatry, University of British Columbia, Vancouver, Canada) – Hypoxia facilitates Alzheimer's disease pathogenesis**

The molecular mechanism underlying the pathogenesis of majority of sporadic Alzheimer's disease (AD) cases is unknown. A history of stroke was found to be associated with development of some AD cases, especially in the presence of vascular risk factors. A reduced cerebral perfusion is a common vascular component among AD risk factors. Hypoxia is a direct consequence of hypoperfusion. We identified a functional hypoxia responsive element (HRE) in BACE1 promoter. Hypoxia increased APP CTFβ production by increasing BACE1 gene transcription and expression in *vitro *and in *vivo*. This paper showed that hypoxia facilitated AD pathogenesis. Under hypoxic condition APP23 mice, Swedish mutant APP transgenic mice, developed more neuritic plaques than normoxic mice. We found that hypoxia deteriorated the memory impairment in APP23 mice. Our results demonstrate that hypoxia facilitates AD pathogenesis and interventions that improve cerebral perfusion might benefit AD patients.

• **Shuzo Sugita (Department of Physiology, University of Toronto, Toronto, Canada) – Molecular mechanism of GTP-dependent exocytosis **

Many secretory cells utilize a GTP-dependent pathway to trigger exocytotic secretion. However, little is currently known about the mechanism by which this may occur. In the present study we attempted to identify the key signaling pathway that mediates GTP-dependent exocytosis. Incubation of permeabilized PC12 cells with soluble RalA GTPase strongly inhibited GTP-dependent exocytosis. A Ral-binding fragment from Sec5, a component of the exocyst complex, showed a similar inhibition. Point mutations in both RalA (RalA^E38R^) and the Sec5 (Sec5^T11A^) fragment which abolish the RalA-Sec5 interaction also abolished the inhibition of GTP-dependent exocytosis. In contrast the RalA and the Sec5 fragment showed no inhibition of Ca^2+^-dependent exocytosis, but cleavage of a SNARE (soluble-*N*-ethylmaleimide-sensitive factor attachment protein receptor) protein by Botulinum neurotoxin blocked both GTP- and Ca^2+^-dependent exocytosis. In stable RalA and RalB double knockdown cells, GTP-dependent exocytosis was severely reduced and was restored upon reintroducing expression of RalA or RalB by transfection. However, Ca^2+^-dependent exocytosis remained unchanged in the double-knockdown cells. Our results indicate that GTP- and Ca^2+^-dependent exocytosis use different sensors and effectors for triggering exocytosis while their final fusion steps are both SNARE-dependent. They also suggest that endogenous RalA and RalB function specifically as GTP sensors for the GTP-dependent exocytosis.

• **Shao-Jun Tang (Department of Neurobiology and Behavior, University of California, Irvine, USA) – Regulation of Activity-Dependent Protein Synthesis in Dendrites**

Protein synthesis in dendrites is essential for long-lasting synaptic plasticity, but little is known about how synaptic activity is coupled to mRNA translation. Using hippocampal neuron cultures and slices, we have investigated the role of glutamate receptors and mTOR signaling in control of dendritic protein synthesis. We find: 1) Specific antagonists of NMDA, AMPA and metabotropic glutamate receptors abolish glutamate-induced dendritic protein synthesis, whereas agonists of NMDA and metabotropic but not AMPA glutamate receptors activate protein synthesis in dendrites; 2) Inhibition of mTOR signaling, as well as its upstream activators, PI3K and AKT, block NMDA receptor-dependent dendritic protein synthesis. Conversely, activation of mTOR signaling induces dendritic protein synthesis; and 3) Dendritic protein synthesis activated by tetanus-mediated LTP induction in hippocampal slices requires NMDA receptors and mTOR signaling. These results suggest critical role of the NMDA receptor-mTOR signaling pathway in regulating protein synthesis in dendrites of hippocampal neurons.

• **Yuanxiang Tao (Department of Anesthesiology and Critical Care Medicine, Johns Hopkins University, Baltimore, USA) – Are the PDZ domains at excitatory synapses potential molecular targets for prevention and treatment of chronic pain?**

The PDZ (Postsynaptic density 95, Discs large, and Zonula occludens-1) domains are ubiquitous protein interaction modules often found among multi-protein signaling complexes at excitatory synapses. In the mammalian central nervous system, C-terminal motifs of N-methyl-d-aspartate (NMDA) receptor subunits NR2A and NR2B bind to the first and second PDZ domains of postsynaptic density (PSD)-95, PSD-93, and synaptic-associated protein (SAP)102, whereas C-terminal motifs of α-amino-3-hydroxy-5-methyl-4-isoxazolepropionic acid (AMPA) receptor subunit GluR2 interacts with the PDZ domain of protein interacting with C-kinase 1 (PICK1) and glutamate receptor interacting protein (GRIP). These PDZ-containing proteins not only are involved in synaptic trafficking of NMDA receptors and AMPA receptors but also couple the receptors to intracellular proteins and signaling enzymes, such as neuronal nitric oxide synthase (nNOS) and protein kinase C (PKC). Recent preclinical research shows that PSD-93, PSD-95, and PICK1 are highly expressed in the dorsal horn of the spinal cord. Immunocytochemical studies demonstrate that their immunoreactivities occur at a higher density in the superficial laminae and at a lower density in other laminae of the spinal dorsal horn. Spinal PSD-93 or PSD-95 deletion prevents NMDA receptor-dependent chronic pain from spinal nerve injury or injection of complete Freund's adjuvant (CFA) without affecting nociceptive responsiveness to acute pain. In addition, the disruption of the PDZ domain-mediated protein interaction between GluR2 and PICK1 in the spinal cord of rats or the knockout of PICK1 in mice has recently been shown to produce antinociceptive effects in AMPA receptor-dependent chronic pain caused by peripheral nerve injury and CFA, with preservation of acute pain transmission. Further studies have demonstrated that PSD-93 or PSD-95 deletion may alter the synaptic NMDA receptor expression and function in spinal cord neurons, which, in turn, may result in impaired NMDA receptor-dependent chronic pain. However, the underlying mechanism by which PICK1 lead to antinociception in chronic pain states is unclear. Our preliminary work indicates that CFA-induced chronic pain might increase time-dependent PKC phosphorylation of GluR2 Ser880, disrupt interaction of GluR2 with GRIP (but not with PICK1), and lead to PKC-dependent internalization of GluR2 (but not of GluR1) in the spinal cord neurons. GluR2 internalization might facilitate Ca2+ permeability and increase AMPAR function and neuronal activity, which may contribute to spinal central sensitization associated with chronic pain states. PICK1 deletion might reduce PKC phosphorylation of GluR2 Ser880 by blocking the recruitment of PKC to synaptic GluR2 and decrease PKC-dependent internalization of GluR2 in spinal cord neurons, which, in turn, might result in blunted AMPA receptor-dependent central sensitization in chronic pain. Therefore, it is very likely that PDZ domains at excitatory synapses may be new molecular targets for prevention and treatment of chronic pain.

• **Yu Tian Wang (Brain Research Center, University of British Columbia, Vancouver, Canada) – Synaptic plasticity in learning and memory**

Synaptic plasticity (i.e. a dynamic change in the strength of synaptic transmission between neurons), such as long-term potentiation (LTP) and depression (LTD) observed at the glutamatergic synapses of the CA1 region of the hippocampus, has long been proposed as a primary cellular mechanism for learning and memory. However, evidence for a definitive role of either LTP or LTD in learning and memory remains missing due to the lack of a specific inhibitor for LTP or LTD. Evidence accumulated in recent years strongly suggests that AMPA subtype glutamate receptors (AMPARs) are continuously cycling between the plasma membrane and intracellular compartments via vesicle-mediated plasma membrane insertion and endocytosis, and that facilitated AMPAR insertion and endocytosis at postsynaptic membranes contributes to the expression of LTP and LTD, respectively. Using a combination of recombinant receptor expression systems and hippocampal brain slice preparations, we were able to demonstrate that facilitated endocytosis of postsynaptic AMPARs during LTD is AMPAR GluR2 subunit-specific. These studies have lead us to develop a GluR2-derived interference peptide that, when delivered into neurons in the brain, can specifically block the expression of LTD without affecting normal basal synaptic transmission in many regions of the brain. Using the membrane-permeant form of the GluR2 peptide as a specific inhibitor of LTD, we were able to probe the role of LTD in freely moving rats with unprecedented specificity, and thereby provide evidence for the involvement of LTD in a number of learning and memory-related behaviours. Our work not only provides the first evidence for a definitive role of LTD in learning and memory, but also demonstrates the utility of peptides that disrupt AMPAR trafficking, the final step in the expression of synaptic plasticity, as tools to examine the critical role of LTD and/or LTP in specific aspects of learning and memory in conscious animals.

• **Newton Woo (NICHD, NIH, Bethesda, USA) – Regulation of Bi-directional Plasticity by BDNF**

Initially characterized for its role in neuronal survival and differentiation, *B*rain *D*erived *N*eurotrophic *F*actor (BDNF) has emerged as a key regulator of synaptic plasticity, which is a persistent change in synaptic strength thought to underlie many cognitive functions. A salient characteristic of this ubiquitously expressed neurotrophin is that expression of BDNF is activity dependent, which has profound implications in development and neuronal plasticity. BDNF is synthesized as a precursor (proBDNF) that can undergo proteolytic cleavage to yield mature BDNF (mBDNF). Initially, the biological actions elicited by neurotrophins, including BDNF, were thought to only arise from the processed mature form. However, recent groundbreaking studies have demonstrated distinct biological roles for several proneurotrophins and their mature form via distinct receptor/signaling cascades. This highlights the importance of the conversion of pro- to mature protein as a key regulatory step in neurotrophin actions. However, whether this proteolytic cleavage plays a role in synaptic plasticity has not been previously addressed. Here, I present evidence that such a conversion process from pro- to mature BDNF is important for one long-lasting form of synaptic plasticity, namely late-phase LTP (L-LTP). Application of strong theta-burst stimulation (TBS) induces L-LTP that is protein synthesis dependent. In BDNF +/- mice, L-LTP is significantly impaired after TBS simulation. However, L-LTP can be rescued in BDNF +/- mice when hippocampal slices were preincubated with mBDNF but not proBDNF. Subsequent experiments identified the tPA/plasminogen system plays a critical role for both BDNF processing and L-LTP expression in the mouse hippocampus. To investigate the location of this conversion process, we performed several additional experiments using cleavage specific antibodies. It was discovered that in cultured hippocampal neurons, low frequency stimulation triggered proBDNF secretion, whereas high frequency stimulation induced the expression of mBDNF. Strikingly, tPA secretion only occurred after high frequency stimulation. Moreover surface staining of mBDNF was greatly enhanced upon depolarization. These results suggest that neuronal activity regulates the ratio of extracellular pro- to mature BDNF via tPA secretion. Finally, I present data that proBDNF facilitates hippocampal long-term depression (LTD). This facilitation of NMDAR LTD is dependent on the p75 neurotrophin receptor (p75^NTR^). Mice that lack p75^NTR ^exhibit a selective impairment in the NMDA-dependent form of long-term depression (LTD), but display normal expression of other hippocampal forms of synaptic plasticity. This selective deficit may be the result of a significant reduction in NR2B, a NMDA receptor subunit uniquely involved in LTD. Activation of p75^NTR ^by proBDNF enhanced hippocampal LTD. Our results challenge the classic view that the processed neurotrophins is the only functional form of neurotrophins to elicit biological actions and that an unexpected function of p75^NTR ^is to regulate the expression of hippocampal synaptic depression. Taken together, these results suggest a universal "Yin-Yang" model where pro- and mature- BDNF play diametrically opposite roles in synaptic plasticity.

• **Melanie A Woodin (Department of Cell and Systems Biology, University of Toronto, Toronto, Canada) – Bidirectional spike-timing dependent plasticity of inhibitory transmission in the hippocampus**

The mammalian hippocampus, owing to its crucial role in memory, has been the primary focus of research into synaptic plasticity. Most studies have examined plasticity at excitatory (glutamatergic) synapses, despite the fact that neuronal output is not determined by the level of excitatory transmission alone, but by the levels of coincident excitatory and inhibitory transmission. In this study, we examined spike-timing dependent plasticity of GABAA receptor mediated inhibitory transmission in area CA1 of hippocampal slices from mature rats (6–8 weeks). Because the amplitude and reversal potential of GABAR currents are largely determined by intracellular chloride concentration, we first determined the GABAR reversal potential under conditions of intact intracellular chloride using the permeating agent gramicidin. Surprisingly, we found that GABAR reversal potential was ~12 mV hyperpolarized compared to the reversal potential in a previous study of STDP of GABAR mediated transmission in P12-19 slices, as well as to our own recordings from P12-19 slices. We then performed a series of whole-cell recordings to determine the intacellular chloride concentration necessary to reproduce the GABAAR reversal potential measured with gramicidin. This allowed us to employ long-term, stable whole-cell recording to investigate whether a spike-timing protocol could induce changes in GABAAR reversal potential. Surprisingly, pairing of presynaptic stimulation with postsynaptic spiking led to bidirectional changes in the reversal potential, with the direction of change being dependent on the interval between pre- and post stimulation. When the postsynaptic neuron was made to fire bursts of action potentials 5 ms after presynaptic stimulation (correlated), at 5 Hz for 90 seconds, a depolarization of the reversal potential was seen. However, when the interval was lengthened to 100 ms (uncorrelated), a hyperpolarization of the reversal was seen. Due to the interplay between excitatory and inhibitory transmission, we suggest that this form of GABAergic plasticity may contribute to the enhancement of excitatory transmission under certain conditions.

• **Zhen Yan (Department of Physiology and Biophysics, State University of New York at Buffalo, Buffalo, USA) – Interactions between Acetylcholine, Amyloid and Ion Channels in Alzheimer's Disease**

It has been well recognized that one prominent feature of Alzheimer's disease (AD) is the accumulation of b-amyloid (Ab), a major component of senile plaques. Another fundamental feature of AD is the severe degeneration of basal forebrain (BF) cholinergic neurons and deficient cholinergic functions in prefrontal cortex (PFC), a brain region implicated in high-level cognitive processes. We have found that cholinergic inputs from BF, by activating M1 muscarinic receptors in PFC pyramidal neurons, regulate the GABAA receptor channel, a key player in working memory, through a PKC/Src-dependent mechanism. The M1 regulation of GABA transmission in PFC is impaired in the APP transgenic model of AD, due to the Ab interference with M1 activation of PKC. On the other hand, glutamate inputs from PFC, by activating Group III metabotropic glutamate receptors (mGluRIII) in BF neurons, suppresses NMDAR currents through an actin-dependent mechanism. Ab selectively disrupts mGluRIII regulation of NMDAR channels in BF cholinergic neurons, which may due to their sensitivity to Ab-induced cytoskeleton disintegration. Thus, our results have provided a potential mechanism for the synaptic failure of PFC pyramidal neurons and the selective degeneration of BF cholinergic neurons at the early stage of AD.

• **Megumu Yoshimura (Department of Basic Medicine, Kyushu University, Kyushu, Japan) – Synaptic mechanisms of acupuncture in the spinal dorsal horn revealed by in vivo patch-clamp recordings**

According to Chinese literatures, more than 300 acupoints have been described. Stimulation of each point elicits certain analgesia in the corresponding area. Physiological examinations have been made to unveil the mechanisms of the analgesic action of acupuncture, however, clear results have not been provided, because of difficulty in how to approach the changes of nociceptive transmission in CNS. One of promising approaches will be an in vivo patch-clamp recording from spinal dorsal horn neurons to see what is happening during acupuncture. Thus, we applied the in vivo patch-clamp recordings from substantia gelatinosa neurons that receive noxious inputs and observed a change in excitatory and inhibitory synaptic currents occuring spontaneously or evoked by stimulation of the skin in the receptive field. To enhance the nociceptive inputs from periphery, we used CFA induced inflammatory rats injected right hind paw. In this chronic pain model, spontaneous EPSCs with higher magnitude were observed in the majority of SG neurons. Application of acupuncture to the contralateral ST36 near the knee joint did not affect the spontaneous EPSCs. However, large amplitude of spontaneous IPSCs were elicited with the frequency of 2 to 10 Hz. Next, we tested the cell firing in the SG by stimulation of the skin with toothed forceps during the acupuncture. The skin-evoked spike firing was effectively inhibited reversibly by the acupuncture. In our previous slice experiments, noradrenaline and serotonin increased spontaneous IPSCs with large ampitude. Other possible candidates responsible for the depression of nociceptive inputs to the SG, such as dopamine, enkephalin, other opioids, substance P, CGRP did not increase the frequency and amplitude of spontaneous IPSCs.

• **Ming Xu (Department of Anesthesia and Critical Care, University of Chicago, Chicago, USA) – Molecular Mechanisms of neuronal plasticity induced by drugs of abuse**

Drug addiction is a brain disease that is characterized by the compulsive seeking and taking of a drug despite known adverse consequences. Drug addiction is also long-lasting with a high propensity to relapse. The brain dopaminergic system is a key neural substrate for mediating the actions of abused drugs. The development of drug addiction is thought to involve coordinated temporal and spatial actions of specific dopamine receptors, signaling molecules, and target molecules that change synaptic reorganizations in the brain. To dissect mechanisms underlying drug-induced neuroadaptations, we have made and analyzed D1 receptor mutant mice. We found that the D1 receptor mediates the locomotor-stimulating and rewarding effects of cocaine. The D1 receptor also mediates cocaine-induced changes in neuronal dendritic remodeling, ERK and CREB signaling, chromatin remodeling, and gene expression including c-*fos *and AP-1-regulated target genes. These results suggest that the D1 receptor is a major cell surface mediator for drug-induced behaviors and neuroadaptations, and that c-*fos-*regulated gene expression may contribute to the persistent nature of drug-induced behaviors. To investigate intracellular mechanisms of cocaine-induced persistent changes within D1 receptor-expressing neurons, we made D1 receptor neuron-specific c-*fos *mutant mice. We found that c-Fos contributes to the development and extinction of cocaine-induced conditioned place preference and behavioral sensitization, changes in dendritic reorganization, and regulation of immediate early genes and the expression of two classes of target genes that are involved in neurotransmission and neuronal connections. Noticeably, mutations of the D1 receptor gene and c-*fos *share several common consequences after repeated cocaine injections. Together, these findings suggest that the dopamine D1 receptor and c-Fos are a key receptor signaling system that contributes to persistent neuroadaptations to cocaine.

• **Zao C Xu (Department of Anatomy and Cell Biology, Indiana University School of Medicine, Indianapolis, USA) – Synaptic plasticity in pathological conditions**

Synaptic plasticity occurs during development and participates in physiological functions in adulthood during learning and memory. It has also been shown in pathological conditions such as epilepsy. To investigate the synaptic plasticity in neurodegenerative disorders and the underlying mechanisms, synaptic transmission and morphological changes were studied in neurotransplantations after excitotoxic lesion and in neurons after transient cerebral ischemia.

For transplantation studies, striatal primordium were collected from 16 d embryos and implanted into the striatum of adult Sprague-Dawley rats two days after kainic acid lesion. Intracellular recording in vivo and anterograde tracing experiments were performed 2–6 months after the transplantation. For ischemia studies, transient global ischemia was induced in adult Wistar rats. Electrophysiological recording and morphometry analysis of intracellularly stained neurons were performed at different intervals after ischemia.

Spontaneous synaptic activities were greatly reduced in striatal grafts. Cortical or thalamic stimuli elicited monosynaptic excitatory postsynaptic potentials (EPSPs) from neurons in the graft. A late postsynaptic potential (L-PSP) was evoked from many graft neurons (17/27) in addition to the initial EPSPs. Bursting action potentials were generated from the L-PSPs. Light and electron microscopic studies showed that the number of cortical and thalamic afferent fibers significantly reduced in the grafts. Some of these fibers formed dense clusters of terminals making multiple synapses on individual spines and dendrites. L-PSPs also could be evoked from neurons in the striatum and hippocampus following cerebral ischemia. Furthermore, the initial EPSPs were potentiated in ischemia-vulnerable neurons (spiny neurons in the striatum and CA1neurons in the hippocampus) but depressed or unchanged in ischemia-resistant neurons (large aspiny neurons in the striatum and CA3 neurons in the hippocampus) after ischemia. Quantitative analysis of 3-D reconstructed CA1 pyramidal neurons indicated that the total dendritic length in apical dendrites was significantly increased at 24 h after ischemia but remained about the same in basal dendrites. Such increase was due to the dendritic sprouting rather than dendritic extension, which occurred mainly in the middle segment of the apical dendrites.

These results demonstrate that the synaptic plasticity changes also occur in acute neurodegenerative disorders. The plasticity changes in striatal grafts might be the compensatory responses, whereas those in ischemic neurons might be associated with the selective neuronal damage after ischemic insults.

• **Xia Zhang (Department of Psychiatry, University of Saskatchewan, Saskatoon, Canada) – Cannabinoid addiction and cannabinoid medicine**

A. Suppression of cannabinoid rewarding effects and cannabinoid withdrawal syndrome respectively by the interfering peptide Tat-3L4F and lithium

Cannabinoids or marijuana is the most commonly used illicit drug in developed countries. The lifetime prevalence of marijuana dependence is the highest of all illicit drugs in the USA, but there is no effective medication available for treating marijuana addiction. Our recent two studies show potential strategies for treating marijuana addiction in humans. In the first study we found a physical interaction of the enzyme PTEN with a region in the third intracellular loop (3L4F) of 5-HT2C receptor (5-HT2cR) in cell cultures. PTEN limits agonist-induced phosphorylation of 5-HT2cR through its protein phosphatase activity. We then found the probable existence of PTEN:5-HT2cR complexes in putative dopaminergic neurons in the rat ventral tegmental area (VTA), a brain region in which virtually all abused drugs exert rewarding effects by activating its dopamine neurons. We next synthesized the interfering peptide Tat-3L4F, which is able to disrupt PTEN coupling with 5-HT2cR. Tat-3L4F or the 5-HT2cR agonist Ro600175 suppressed the increased firing rate of VTA dopaminergic neurons induced by delta9-tetrahydrocannabinol (THC), the psychoactive ingredient of marijuana. Using behavioral tests, we observed that Tat-3L4F or Ro600175 blocks conditioned place preference of THC, and that Ro600175, but not Tat-3L4F, produces anxiogenic effects, penile erection, hypophagia and motor functional suppression. These results suggest a potential strategy for treating cannabinoid addiction with the Tat-3L4F peptide. In the second study we demonstrate that lithium treatment prevented the cannabinoid withdrawal syndrome (CWS) in rats, which was accompanied by expression of the cellular activation marker Fos in oxytocin-immunoreactive neurons and a significant increase in oxytocin mRNA expression in the hypothalamic paraventricular and supraoptic nuclei. Lithium also significantly increased blood oxytocin levels. We suggest that the effects of lithium against the CWS are mediated by oxytocinergic neuronal activation and subsequent release and action of oxytocin within the CNS. This hypothesis is supported by further findings that the effects of lithium against the CWS were antagonized by an oxytocin antagonist and mimicked by oxytocin. These results led us to conduct a small-scale, pilot clinical study showing positive therapeutic effects of lithium against the CWS in patients with pure cannabinoid addiction.

B. Promotion of hippocampal neurogenesis and suppression of anxiety and depression by cannabinoids. The adult hippocampus contains neural stem/progenitor cells (NS/PCs) capable of generating new neurons, i.e., neurogenesis. Most drugs of abuse examined to date decrease adult hippocampal neurogenesis, but the effects of cannabinoids remain unknown. We show that both embryonic and adult rat hippocampal NS/PCs are immunoreactive for CB1 cannabinoid receptors. We then found that both the synthetic cannabinoid HU210 and an endogenous cannabinoid promote proliferation, but not differentiation, of cultured embryonic hippocampal NS/PCs likely via a sequential activation of CB1 receptors, Gi/o proteins, and ERK signaling. Chronic, but not acute, HU210 treatment promoted adult hippocampal neurogenesis and exerted anxiolytic- and antidepressant-like effects. X-irradiation of the hippocampus blocked both the neurogenic and behavioral effects of chronic HU210 treatment, suggesting that chronic HU210 treatment produces anxiolytic- and antidepressant-like effects likely via promotion of hippocampal neurogenesis.

• **Mei Zhen (Department of Medical Genetics and Microbiology, University of Toronto, Toronto, Canada) – SAD kinase regulates neuronal polarity and synapse formation**

Using *C. elegans *GABAergic neurons as a model system, we identified a Ser/Thr kinase SAD-1 as a key player in establishing axon/dendrite polarity and synaptic structures. In *C. elegans *the loss of the SAD-1 function leads to the accumulation of synaptic vesicles at dendritic regions of neurites, furthermore synaptic vesicles are loosely clustered at chemical synapses. Using genetic and biochemical approaches, we determined two separate genetic pathways through which SAD-1 kinase functions: During early differentiation of neurons, SAD-1 physically interacts with a scaffolding protein Neurabin to restrict the axonal fate of the developing neurites. After the establishment of axon and dendrites, SAD-1, restricted at presynaptic region by several presynaptic channels, negatively controls the incorporation of active zone proteins at chemical synapses. We are further delineating the activator and downstream effectors of the SAD kinase.

• **Min Zhuo (Department of Physiology, University of Toronto, Toronto, Canada) – Cortical potentiation and its roles in persistent pain and fear**

Neuronal synapses in the central nervous systems are plastic, and can undergo long-term changes throughout life. Studies of molecular and cellular mechanisms of such changes not only provide important insight into how we learn and store new knowledge in our brains, but also reveal the mechanisms of pathological changes occurring following a noxious stimulus such as pain and fear. Using integrative approaches including genetic, pharmacological, electrophysiological and behavioral studies, we explore the synaptic mechanisms for LTP and LTD in the cingulate and prefrontal cortex of adult mice. We found that activation of postsynaptic NMDA receptor is required for the induction of synaptic LTP. The expression of cingulate LTP is likely mediated by postsynaptic AMPA receptors, while presynaptic form of paired-pulse facilitation remained unchanged during synaptic potentiation. Activation of calcium-calmodulin stimulated adenylyl cyclase AC1 is required for the induction of LTP. Similar to the hippocampus, NMDA NR2A subtype receptor is required for the induction of LTP. NMDA NR2B receptors, however, also contribute to synaptic potentiation. Genetic reduction of NR2B expression or pharmacological inhibition of NR2B receptor by selective antagonists reduced behavioral contexual fear memory. The possible contribution of the ACC to the formation of fear memory is its role in pain perception. Supporting this hypothesis, inhibition of NMDA NR2NB receptors in the ACC inhibited behavioral sensitization to non-noxious stimuli. Our results provide strong evidence that synaptic potentiation within the cingulate/prefrontal cortex play important roles physiological and pathological responses to noxious sensory stimuli and injury, including emotional fear and persistent pain.

## Competing interests

The author(s) declare that they have no competing interests.

## Authors' contributions

Each author provided abstract for the 1st International Conference on Synapse, Memory, Drug Addiction and Pain, as indicated in the manuscript. MZ collected and organized abstracts for publishing. All authors read and approved the final manuscript.

